# Role of tumor microenvironment in the pathobiology of ovarian cancer: Insights and therapeutic opportunities

**DOI:** 10.1002/cam4.1741

**Published:** 2018-08-21

**Authors:** Alia Ghoneum, Hesham Afify, Ziyan Salih, Michael Kelly, Neveen Said

**Affiliations:** ^1^ Department of Cancer Biology Wake Forest University School of Medicine Winston Salem North Carolina; ^2^ Department of Pathology Wake Forest University School of Medicine Winston Salem North Carolina; ^3^ Department of Obstetrics and Gynecology Wake Forest University School of Medicine Winston Salem North Carolina; ^4^ Department of Urology Wake Forest University School of Medicine Winston Salem North Carolina

## Abstract

Ovarian cancer is the fifth most common cancer affecting women and at present, stands as the most lethal gynecologic malignancy. The poor disease outcome is due to the nonspecific symptoms and the lack of effective treatment at advanced stages. Thus, it is of utmost importance to understand ovarian carcinoma through several lenses and to dissect the role that the unique peritoneal tumor microenvironment plays in ovarian cancer progression and metastasis. This review seeks to highlight several determinants of this unique tumor microenvironment, their influence on disease outcome and ongoing clinical trials targeting these determinants.

## 1 INTRODUCTION

Epithelial Ovarian cancer (OvCa) is the leading cause of death from gynecologic malignancies in the United States.^1^ More than 75% of patients are diagnosed at late stages due to the incipient protracted nature of the disease and lack of specific diagnostic symptoms and/or biomarkers.^2^ Despite aggressive surgical debulking and cytoreduction, 80% of patients experience recurrence with limited treatment options and poor survival.^1^ Indeed, optimal surgical debulking (<1 cm of residual tumor) significantly improves patients’ survival compared to suboptimal debulking^3^ (>1 cm of residual tumor) due to widespread microscopic and inaccessible lesions throughout the abdomen.^3^

High‐grade serous cancer (HGSC) is the most common subtype (∼70%) and accounts for the majority of deaths.^1^, ^2^ HGSC was long believed to arise from the ovarian surface epithelium (OSE) or ovarian inclusion cysts. Recent studies suggest that a substantial proportion of cases arise from precursor lesions in the fallopian tubal epithelium (FTE).^4^, ^5^ Other pathological subtypes of OvCa include endometrioid, clear cell, and mucinous.^2^ Irrespective of the cell of origin or pathological subtype, OvCa preferentially metastasizes to the peritoneal cavity.^6^ The dynamic interaction of the transformed cells with the unique peritoneal tumor microenvironment (TME) not only influences tumor progression, but also results in the evolution of other genetic, and epigenetic events that deeply impact disease outcome and response to therapy. The lack of success in effectively eradicating OvCa can be attributed to the complex interconnected signaling networks coupled within the distinctive peritoneal TME.^6^ Therefore, understanding the pathobiology of OvCa and the unique TME that hosts this malignancy is crucial in development of more sensitive diagnostic, prognostic and therapeutic tools.

### 1.1 Determinants of peritoneal metastasis

1. *OvCa cells* are unique among cancers that they have diverse progenitors that express common epithelial markers as keratins, EpCAM and E‐cadherin as well as mesenchymal markers as vimentin and N‐cadherin.^7^ Malignant cells are shed from the primary tumor into the peritoneal cavity survive as free‐floating single cells or spheroids in the “malignant ascitic fluid” that is encountered in the majority of patients with OvCa.^6^, ^8^ Single cells and spheroids can survive anchorage‐independent apoptosis “anoikis,” proliferate in suspension and seed onto the mesothelial lining of the peritoneal cavity, resulting in extensive peritoneal dissemination.^9^ Malignant cells isolated from ascitic fluid exhibit dual “hybrid” as well as heterogeneous E‐and N‐Cadherin expression.^9^ This cadherin‐plasticity influences cell‐cell interactions, spheroid formation, and is implicated in the dynamic switch between epithelial‐mesenchymal transition (EMT) and mesenchymal‐epithelial transition (MET). EMT‐MET switch is regulated by sequential transcriptional machinery with early induction of the transcription factors SNAIL (*SNAI1*); followed by *SNAI2* (*SLUG*), *ZEB1/2* and *TWIST*.^10^, ^11^, ^12^, ^13^ EMT‐transcription factors are induced by a plethora of upstream factors that act individually or synergistically to induce an OvCa invasive phenotype. In addition to intrinsic EMT inducers activated in cancer cells, cues from the peritoneal TME strongly induce EMT.^14^, ^15^, ^16^, ^17^ The expression of EMT‐inducing transcription factors is associated with metastatic, recurrent, and chemo‐resistant tumors.^3^, ^10^, ^18^ The correlation between EMT and aggressiveness of OvCa is supported by E‐cadherin downregulation^19^ and overexpression of mesenchymal signatures specifically transforming growth factor‐beta and its receptors (TGFβ/TGFβRs), CD44,^20^ bone morphogenetic proteins and their receptors (BMPs/BMPRs), receptor tyrosine kinases and their ligands,^13^ Wnt^21^, ^22^ and Notch^12^ signaling pathways.
2. *Mesothelial cells* are organized single layer of simple squamous epithelium covering submesothelial extracellular matrix (ECM) rich in collagen I.^23^, ^24^, ^25^ The propensity of OvCa to metastasize to the mesothelial cells is initially instigated by cancer cell secretome that preconditions the mesothelial cell niche, inducing the expression of pro‐inflammatory mediators as bioactive lipids, cytokines/chemokines,^26^, ^27^, ^28^ ECM/integrins,^24^, ^29^, ^30^, ^31^ cell adhesion molecules as VCAM1, ICAM1, CD44/HA,^32^, ^33^, ^34^ and uPA/uPAR.^35^, ^36^ The bidirectional cross‐talk between cancer and mesothelial cells activates multiple downstream signaling pathways that corroborate to promote cancer cell colonization, mesothelial clearance, and invasion of the submesothelial layers.^37^, ^38^, ^39^
3. *The omentum* is a double‐layered peritoneal fold that covers the intestines and abdominal organs. Physiologically, it functions as a fat and energy depot due to the abundance of white adipocyte.^40^, ^41^ The bidirectional interaction between omental adipocytes and cancer cells is instigated by cancer cell secretome inducing dedifferentiation and reprogramming of adipocytes into a pre‐adipocyte/fibroblastoid stage secreting adipokines,^42^, ^43^ cancer‐associated adipocytes (CAA)^43^ (Figure 1). In this process, lipolysis is induced in adipocytes releasing fatty acids and glycerol. In turn, OvCa cells take up and use fatty acids for generation of energy by β‐oxidation^42^ to meet the increasing demands of the rapidly proliferating cells.

**Figure 1:**
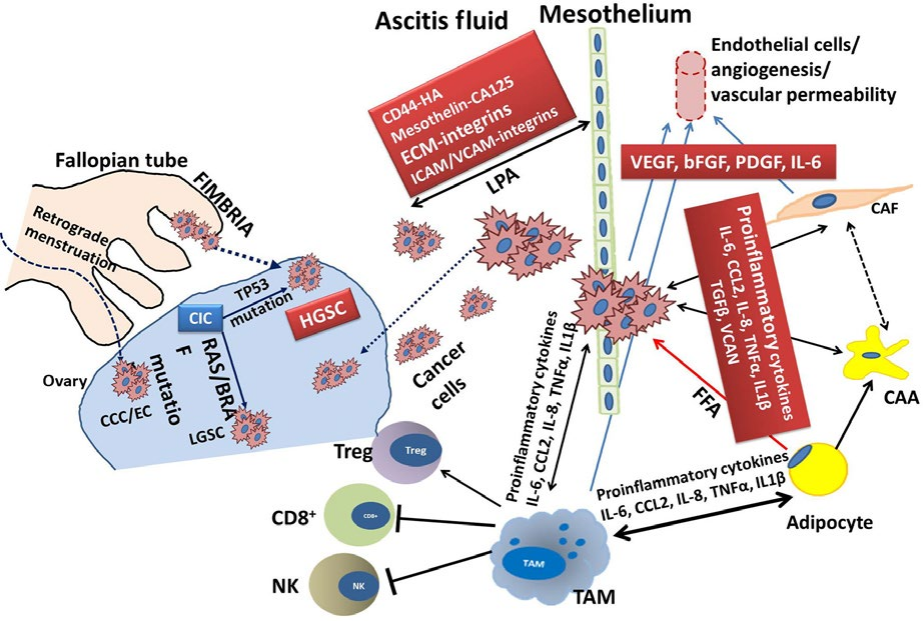
Schematic representation of the key cell types in ovarian cancer microenvironment and the molecules involved in their interactions. HGSC, high‐grade serous cancer; LGSC, low‐grade serous cancer; CCC, clear cell carcinoma; EC, endometrial carcinoma; CIC, carcinoma in situ; CAA, cancer‐associated adipocyte; CAF, cancer‐associated fibroblast; FFA, free fatty acids; VEGF, vascular endothelial growth factor; bFGF, basic fibroblast growth factor; PDGF, platelet‐derived growth factor; VCAN, versican; CD8+, cytotoxic T cell; Treg, regulatory T cell; ECM, extracellular matrix; IL‐x, interleukin‐x; ICAM/VCAM, intercellular/vascular adhesion molecule; HA, hyaluronic acid; CA125, cancer antigen 125; LPA, lysophosphatidic acid; NK, natural killer cell; TAM, tumor‐associated macrophage; TGFβ, growth transforming growth factor β; TNFα, tumor necrosis factor‐α
4. *Cancer‐associated fibroblasts* (*CAFs*). The origin of CAFs in the peritoneal milieu is still enigmatic. Many studies highlighted the significance and the roles of CAFs in OvCa peritoneal spread,^44^, ^45^ response to standard of care therapy, and even proposed the importance of targeting CAF‐derived factors that support OvCa.^44^, ^45^, ^46^, ^47^, ^48^ In the peritoneal milieu, CAFs stem from various origins. The activation of resident fibroblasts and mesenchymal stem cells has been considered the main origin of CAFs.^49^ Mesothelial cells present an important source of activated fibroblasts in inflammatory/fibrotic pathologies as peritoneal dialysis, where they are converted into myofibroblasts through mesothelial to mesenchymal transition (MMT).^50^ In support of this, submesothelial fibroblasts expressed both mesothelial (calretinin, cytokeratins, mesothelin) and myofibroblast (α‐SMA) markers in specimens from patients with peritoneal metastases from ovarian and colon cancers.^51^ Another source of CAFs is omental adipocytes that have undergone delipidation/de‐differentiation.^43^, ^52^, ^53^ This hypothesis was based on reports of adipocyte de‐differentiation into fibroblasts that occur in inflammatory fibrotic changes encountered in dysfunctional adipose tissues in obesity and type‐2 diabetes.^54^ Moreover, the endothelial to mesenchymal transition reported in vasculopathies and atherosclerotic plaques^55^ was suggested as a source of CAFs in OvCa.^56^

The CAF phenotype is induced by TME cues characterized by inflammation, and hypoxia, activating fibroblasts to exhibit characteristics of both myofibroblasts and secretory phenotype.^44^, ^45^, ^46^, ^51^ CAFs can be activated by multiple mechanisms triggered by OvCa cells’ secretome as TGF‐β1, inflammatory cytokines and chemokines, ROS and MMPs,^46^ as well as secreted ECM proteins, the hallmark of myofibroblast phenotype. Transcriptome profiling of microdissected stromal and epithelial components of HGSC and TGF‐β‐treated normal ovarian fibroblasts^45^ revealed TGFβ‐upregulated ECM genes. Functional evaluations in coculture experiments further showed that TGFβ enhanced the aggressiveness of OvCa cells by upregulating versican (VCAN) in CAFs through TGFβ receptor type II (TGFβRII) and SMAD signaling. Consequently, VCAN promoted OvCa cell motility and invasiveness by activating the NF‐κB signaling pathway and by upregulating expression of CD44, matrix metalloproteinase‐9, and the hyaluronan‐mediated motility receptor.^45^ Other secreted ECM proteins upregulated and secreted by CAFs include periostin,^45^, ^57^ secreted phosphoprotein,^57^, ^58^ and cartilage oligomeric matrix protein (COMP).^45^ These secreted ECM proteins, in turn, trigger a plethora of signaling pathways as PI3K‐AKT as well as NFkB that promote OvCa spread, recurrence and chemoresistance.^45^, ^59^, ^60^ In addition, increased number of CAFs was associated with advanced OvCa stage, higher frequency of metastases, and lymphatic and microvessel density.^48^ The findings that the molecular cross‐talk between cancer cells and CAFs in the OvCa TME is regulated by TGFβ/TGFβRs/SMAD pathway in CAFs and triggers multiple oncogenic pathways in OvCa cells warranted the initiation of clinical trials targeting TGFβ/TGFβRs as well as PI3K inhibitors in combination with standard of care therapy (summarized in Table 1).

**Table 1:** Current ongoing clinical trials of therapeutics that target tumor microenvironment, with their corresponding targets and phase in clinical trial

Drug	Target	Clinical trial	NCT trial
Aflibercept (VEGF trap)	VEGF	Phase 2	NCT00327171 NCT00327444 NCT00396591
Bevacizumab + paclitaxel and carboplatin	VEGF‐A	Phase 3	NCT01239732
Bevacizumab and Erlotinib	VEGF‐A + EGFR	Phase 2	NCT00130520
Bevacizumab + Carboplatin	VEGF‐A	Phase 2	NCT00937560 NCT00744718
Chiauranib	Serine‐threonine kinases	Phase 1/2	NCT03166891
Nintedanib + Bevacizumab	VEGFR1/2/3, FGFR1/2/3 and PDGFRα/β	Phase 1	NCT02835833
INCB062079	FGFR4	Phase 1	NCT03144661
Sorafinib + paclitaxel and carboplatin	Multi‐targeted RTKi	Phase 2	NCT00390611
Sunitinib (SU11248)	Multi‐targeted RTKi	Phase 2	NCT00543049 NCT00768144 NCT00453310
Tocilizumab and IFN‐α2b+ Carboplatin and Caelyx or doxorubicin	IL‐6R	Phase 1	NCT01637532
Siltuximab (CNTO 328)	IL‐6R	Phase 2	NCT00841191
Plerixafor	CXCR4	Phase 1	NCT02179970 NCT03277209
PD 0360324 + cyclophosphamide	M‐CSF	Phase 2	NCT02948101
Celecoxib + cyclophosphamide	COX‐1 and COX‐2	Phase 2	NCT00538031
Ketorolac	COX‐1 and COX‐2/GTPase inhibition	Phase 0	NCT02470299
Metformin + paclitaxel and carboplatin	Antidiabetic medication/metabolism	Phase 1 Phase 2	NCT02312661 NCT02437812
Metformin	Antidiabetic medication/metabolism	Phase 2	NCT01579812
Metformin + atorvastatin + doxycycline + mebendazole	Antidiabetic medication/metabolism	Phase 3	NCT02201381
INCAGN01876 + Nivolumab + Ipilimumab	TNFα, PD‐1 and CTLA‐4.	Phase 1/2	NCT03126110
MK‐3475 (pembrolizumab) + Gemcitabine and cisplatin	PD‐1	Phase 2	NCT02608684
Oregovomab and Nivolumab	CA‐125 and PD‐1	Phase 1/2	NCT03100006
Durvalumab (MEDI4736 + motolimod) + pegylated liposomal doxorubicin	PD‐L1 and TLL8	Phase1/2	NCT02431559
Autologous Monocytes + Sylatron (PegIFNα) + Actimmune (IFNγ‐1b)	Immunotherapy	Phase 1	NCT02948426
Vigil bi‐shRNA furin and GMCSF (FANG) Augmented Autologous Tumor Cell Immunotherapy	TGFβ1/TGFβ2 + Immune stimulation	Phase 2	NCT02346747
Vigil (Adjuvant FANG)	TGFβ1/TGFβ2 + Immune stimulation	Phase 2	NCT01309230
Atezolizumab and Vigil	PDL1 and TGFβ1 and TGFβ2	Phase 2	NCT03073525
PI3K and PARP	BKM120 and Olaparib	Phase 1	NCT01623349
PI3K (mutated/amplified) and IGF1R	BYL719 and AMG 479 (ganitumab)	Phase 1b/2	NCT01708161
PI3K	BKM120	Phase 1	NCT01068483
NK immunotherapy	Combination of Cryosurgery and NK Immunotherapy	Phase 2	NCT02849353
Therapeutic autologous Antigen‐Specific CD4^+^ lymphocytes	Immunotherapy	Phase 1	NCT00101257

- *Tumor‐associated macrophages* (*TAMs*) are encountered in the pro‐inflammatory peritoneal TME rich in cytokines/chemokines that recruit macrophages. The cross‐talk between cancer cells and TAMs upregulates the secretion of inflammatory mediators^27^, ^28^, ^61^, ^62^ which influence tumor migration and invasion through activation of NFκB, the key regulator of pro‐inflammatory molecules in TAMs and cancer cells. Increased TAMs not only promotes cancer cell invasiveness but also contributes to immunosuppressive environment suppressing T cells, dendritic (DCs) and natural killer (NK) cells functions.^8^ TAMs also contribute to the phenotypic switch of fibroblasts into CAFs, and in turn activate multiple pathways that lead to chemoresistance, recurrence, and poor prognosis.^63^, ^64^ The augmented inflammatory TME promoted clinical trials targeting inflammatory cytokines/chemokines and their receptors, as well as COX‐2 inhibitors (Table 1).
- *Myeloid‐derived Suppressor Cells* (*MDSCs*) are heterogeneous population of myeloid cells that, in the immature state, are present in the bone marrow and lack suppressive activity. When activated, these cells become potent suppressors of T‐cell function. MDSCs accumulate in tumors in response to growth factors, and inflammatory mediators,^65^ that upregulate CXCR4 and its ligand CXCL12 in cancer‐associated MDSCs providing a rationale for targeting CXCR4 in OvCa therapy^66^ (Table 1). Increased MDSCs in the OvCa TME also maintain OvCa stem cells phenotype.^67^
- *Dendritic cells* (*DCs*) are specialized antigen‐presenting mononuclear cells that in their immature state exhibit phagocytic ability, and when functionally mature, become immune‐stimulatory. However, plasmacytoid (tolerogenic) dendritic cells (PDCs) were reported in malignant ascites of OvCa patients.^68^ DCs are sensitized after exposure to tumor antigen, and stimulate the proliferation of naive T cells to initiate the immune response.^69^ DCs process and present antigens via MHC class I or class II molecules to activate CD8^+^ or CD4^+^ T cells.^68^ Increased number of tumor‐infiltrating DCs correlated with favorable prognosis.^69^ The ability of DCs to process and present antigens and stimulate anti‐tumor immune response promoted clinical trials using DCs vaccines with autologous DCs pulsed with tumor cell lysates for patients with recurrent stage III/IV OvCa.^70^, ^71^
- *Tumor‐associated lymphocytes* (*TILs*) comprise T‐cells, and regulatory T cells (T regs) localized in tumor stroma (stromal TILs) or inside tumor islets (intraepithelial TILs). Intraepithelial TILs play a crucial role in controlling tumor growth. CD8^+^ or CD4^+^T‐lymphocytes recognize cancer antigens or over‐expressed self‐antigens processed by DCs through T‐cell receptors (TCRs).^72^ Upon recognition of tumor antigens by TCR/MHC engagement, activated CD8^+^ cytotoxic T cells (CTLs) directly kill malignant cells by mechanisms including perforin/granzyme secretion and/or FasL/Fas binding. The latter was exclusively found in tumor vasculature and allowed tumor cells to evade immune system.^73^, ^74^ Along with CD4^+^ helper T cells, CD8^+^ CTLs secrete various cytokines/chemokines to direct the activities of other immune cells. Several clinical studies in OvCa, reported positive correlation between patient survival and the presence of intra‐epithelial TILs.^75^ Meta‐analysis of several reports that investigated the prognostic value of TILs in OvCa using the CD8^+^ marker to specifically evaluate CTLs, found that intraepithelial CD8^+^ TILs exhibited a consistent and stronger association with patients’ survival.^76^ In a recent multi‐center trial,^77^ HGSOCs showed the highest infiltration of CD8^+^ TILs that were significantly associated with longer overall survival. A high CD8^+^ TILs infiltration also offered a survival benefit in women with endometrioid and mucinous carcinomas, but not the other histotypes. Among HGSOCs, CD8^+^ TILs were favorable regardless of the extent of residual disease after surgery, standard treatment, or germline BRCA1 (not BRCA2) mutation carriers.^77^

TILs’ function is suppressed by regulatory T cells (Tregs), MDSCs, and TAMs, with their secreted plethora of soluble inhibitory factors.^78^ Suppression of T cell functions occurs through downregulation of MHC molecules and co‐stimulatory ligands, with upregulation of inhibitory receptors like programmed cell death protein ligand‐1 (PD‐L1) on tumor cells and CTL antigen‐4 (CTLA‐4, CD152).^79^ PD‐1/CD279 expression on OvCa cells correlated with poor patients’ survival and reduced CD8^+^ TILs, suggesting that PD‐L1 expression promotes an immunosuppressive TME.^80^ These observations promoted clinical trials targeting of PD1 or PDL‐1 as well as CTLA‐4 in OvCa (Table 1). The efficacy of single or dual blockade of PD‐1 and/or CTLA‐4 synergized with standard of care therapy in OvCa models.^79^, ^81^

*Regulatory T‐cells* (*Treg*) *cells* are T‐cell subpopulation that suppresses the function of activated T‐cells. Tregs are divided into naturally occurring thymus‐generated Tregs with a phenotype of CD4^+^CD25^+^FOXP3^+^ and the adaptive Tr1 Treg and Th3 Tregs with variable CD25 expression. The frequency of Treg cells and TAMs was significantly higher in the OvCa patients than those with benign ovarian tumors.^82^ High frequency of Tregs in OvCa specimens was associated with significantly shorter overall survival time. Mechanistic studies showed that IL‐10 secreted by TAMs increases the frequency of Tregs through activation of Foxp3 during T‐cell differentiation.^82^ Consistently, Treg percentages were significantly higher in patients with OvCa than with benign ovarian tumors (BOT) or healthy controls. Higher percentages of Tregs were found in patients with stage III/IV than stage I/II OvCa.^83^ Interestingly, Treg percentages significantly decreased postoperatively in stage I/II OvCa getting similar to those in BOT patients. However, postoperative Treg percentages in patients with stage III/IV remained higher and correlated with the tumor burden. These studies suggested that Tregs could be used to monitor the immunological status of patients with OvCa.^83^ Patients with OvCa expressed Treg subsets with upregulated CTLA‐4 and downregulated expression of CD28.^84^, ^85^ In vitro induced CD8 Tregs blocked CD4 T‐cells proliferation via TGFβ1 and IFN‐ɣ that not only increase the number of Tregs in peripheral blood of OvCa patients, but also recruit and stimulate Treg tumor infiltration and localization.^86^

*Natural killer cells* (*NK*) are lymphocytes of the innate immune system that target cells with low MHC Class‐I expression including tumor cells through cascades involving perforins/granzymes as well as Fas/FasL.^86^ Tumor cells evade immune‐surveillance via several mechanisms. For instance, MUC16/CA125, a high‐molecular weight mucin overexpressed by OvCa has the ability to inhibit NK cell and downregulating CD16. Blocking ADAM17 maintains CD16 on the cell surface, enhancing CD16‐mediated NK cell killing ability.^87^

- *Endothelial cells* are critical to maintain blood vessel structure, angiogenesis and vascular permeability.^88^ Vascular endothelial growth factor (VEGF) has been long identified as the key regulator of angiogenesis and vascular permeability and is produced by cancer and stromal cells.^89^ VEGF contributes to the development of peritoneal carcinomatosis with malignant ascites.^90^ Preclinical and clinical studies showed that VEGF levels inversely correlate with disease prognosis and patients’ survival.^89^, ^91^ VEGF inhibition suppresses tumor growth, dissemination, and ascites production. These findings promoted clinical evaluation and approval of agents targeting VEGF/VEGFRs in patients with OvCa as single agents or in combination with standard of care therapy. Deregulation of normal endothelium in the peritoneal TME is also induced by proangiogenic and pro‐inflammatory factors, bioactive lipids and neuroendocrine hormones produced by OvCa and stromal cells in the peritoneal TME.^89^, ^91^ This upregulation of the proangiogenic factors and their interconnected signaling pathways not only contributes to increased vascular permeability, tumor growth, and angiogenesis, but also contributes to the suboptimal response to standard of care therapy.^89^, ^91^ Therefore, clinical trials targeting these proangiogenic factors, and their receptors in OvCa patients are currently underway (Table 1).
- *Ascitic fluid* develops due to increased vascular and mesothelial permeability with transudation of high‐protein fluid from intravascular compartment to peritoneal cavity in OvCa patients. The oncogenic signals generated from growing tumors, concentrate in ascites, and dynamically change according to the disease subtype, stage, and grade, as well as among patients. The heterogeneity in ascites constituents and their relative concentrations is exemplified by the presence of both oncogenic and tumor suppressive factors. In HGSC, ascites promotes tumor invasiveness and survival and inhibits apoptosis leading to chemoresistance.^92^ Along with the high protein concentration, increased inflammatory cytokines and chemokines and reduced lymphatic flow also contribute to the buildup of ascitic fluid and maintenance of an immunosuppressive TME that impairs the functions of innate and adaptive immune responses.^89^, ^93^, ^94^ Ascites is rich in bioactive lipids as lysophosphatidic acid (LPA), that has been long identified as an OvCa promoting factor.^95^ LPA is produced by OvCa cells, as well as the other cellular components in the peritoneal TME. High levels of LPA in ascitic fluid lead to aberrant receptor signaling with activation of pro‐inflammatory and pro‐survival pathways as well as transactivation of receptor tyrosine kinases, that in turn, contribute to increased production of LPA, growth factors, cytokines/chemokines,^96^, ^97^ further OvCa progression, and are associated with poor prognosis.^8^, ^98^ Other studies^99^, ^100^ reported significantly higher plasma LPA levels in patients with OvCa compared with controls with no ovarian pathology or patients with benign ovarian tumor. Plasma LPA levels significantly associated with disease stage but not with the histological subtype or grade of ovarian cancer. The study suggested that plasma LPA level can be a useful marker for ovarian cancer.^99^, ^100^ The levels of IL‐6, IL‐10 and osteoprotegerin (OPG) in ascitic fluid of HGSC patients were significantly higher in women with advanced disease^101^ and could distinguish EOC from benign controls.^101^ Furthermore, exosomes have been reported in OvCa ascitic fluid as 30‐100 nm micro‐vesicles segregating lipids, proteins, and nucleic acids, within the membrane‐covered vesicles.^102^ Exosomes transfer information between cells to alter gene expression in recipient cells and were found to contain distinct subsets of disease‐specific biomarkers.^103^ At the cellular level, ascitic fluid contains floating cancer cells (as single cells and spheroids), macrophages and immune cells; all contribute to malignant aggressive phenotype of OvCa.^89^, ^91^

Ascitic fluid contains secreted factors produced by the various cells in the peritoneal TME, yet, its utility for diagnosis and/or patient stratification for therapy is still limited. Factors enriched in the malignant ascitic fluid as VEGF, IL‐6, IL‐8, MMPs, and LPA, lack sensitivity, and specificity in OvCa. Combined detection of tumor markers in serum and ascites may improve their diagnostic/prognostic value. However, since ascitic fluid contains floating tumor and immune cells, it can serve as a reliable source for isolation of these cells for autologous immunotherapy. In addition, ascites‐derived cancer cells could be used for generation of patient‐derived xenografts for further characterization and therapeutic screening.

## 2 TREATMENT

Initial treatment options are primary debulking surgery followed by chemotherapy or neoadjuvant chemotherapy followed by surgery. Standard chemotherapy involves carboplatin and paclitaxel. Various targeted therapies are being studied in combination with carboplatin/paclitaxel (Table 1). In addition to the FDA approved targeted therapies as poly (ADP‐ribose) polymerase PARP inhibitors and VEGF inhibitors, other targeted therapies currently in clinical trials include inhibitors of angiogenesis (VEGF/VEGFRs, FGFRs, PDGFRα/β), multi‐target receptor tyrosine kinase (RTKi), Cox‐2, and cytokines and their receptors.

Recently, immunotherapy for advanced OvCa was introduced in clinical trials using immune checkpoint inhibitors targeting PD1, PDL1, and CTL4 to restore the ability of CTLs to eradicate tumor cells. Personalized therapy with autologous tumor and immune cells reprogrammed *ex‐vivo* to stimulate the immune system and overcome immune evasion of OvCa cells are in clinical trials. Moreover, targeting tumor metabolism has recently gained more appreciation evidenced by clinical trials of metformin in advanced HGSC either alone or in combination of standard of care therapy (Table 1).

## 3 CONCLUSION

OvCa carries the largest burden of disease mortality among gynecologic malignancies. Despite initial response to first‐line therapy, recurrence occurs within 18 months. Indeed, successful treatment of OvCa can be achieved by improving our understanding of the complex interplay of cancer cells within the unique peritoneal TME. Several lines of targeted drugs have improved progression‐free survival in some patients with OvCa. For example, patients with ascites would benefit from VEGF targeted therapy. Patients with high intra‐tumoral CD8^+^ TILs or increased CD8^+^ TILs in the ascitic fluid would benefit from immunotherapy or tumor vaccines. In addition, patients with amplified PI3K or harboring activating mutation of *PIK3ca* would benefit from PI3K inhibitors recently introduced in clinical trials (Table 1). Moreover, the growing appreciation of therapeutic efficacy of metformin in OvCa patients, highlight the importance of targeting metabolic programming in OvCa. The strategies outlined in this review as well as the ongoing clinical trials are promising for improving the efficacy of TME‐targeted therapeutics to improve disease outcome and patient quality of life.

## CONFLICT OF INTEREST

None declared.
